# Development and validation of the leadership learning agility scale

**DOI:** 10.3389/fpsyg.2022.991299

**Published:** 2022-12-23

**Authors:** Sophie I. M. Bouland-van Dam, Janneke K. Oostrom, Paul G. W. Jansen

**Affiliations:** Department of Management & Organization, School of Business & Economics, Vrije Universiteit Amsterdam, Amsterdam, Netherlands

**Keywords:** learning agility, leadership, scale development, construct validity, empirical article

## Abstract

**Introduction:**

Learning agility is key in the selection and development of future leaders. However, prior research has failed to clearly conceptualize learning agility and to empirically clarify its dimensions.

**Method:**

We developed the Leadership Learning Agility Scale (LLAS) by using a combination of both deductive and inductive approaches and established scale development and validation procedures. We administered the LLAS among three independent samples of workers and leaders (*N* = 907; *N* = 196; *N* = 219).

**Results:**

Our results indicate that our 18-item LLAS measures the willingness to learn from social experiences, and the drive to apply those lessons in new and challenging leadership roles, and comprises a Developing Leadership, Seeking Feedback, and Developing Systematically dimension. Furthermore, the LLAS showed adequate internal consistency. Leadership learning agility was positively related to achievement motivation, extraversion, and conscientiousness but unrelated to openness to experience.

**Discussion:**

We provided a new scale to measure leadership learning agility that can be applied in both research and practitioner settings.

## Introduction

It is not the most intellectual of the species that survives, or the strongest, but the one that is able best to adapt and adjust to the changing environment (Megginson, 1963).

In order to compete more successfully in today’s complex and unpredictable business context, employees need to be versatile and willing to learn new skills and behaviors to effectively operate in continuously changing business environments (Pulakos et al., 2019; Milani et al., 2021). A concept that is associated with dealing with unexpected changes in business environments is learning agility (Lee and Song, 2022). The meaning in use of learning agility is “to effectively learn and to apply prior learnings” (e.g., Swisher, 2014). Although learning agility is essential for many types of employees, it generally has been linked to leaders’ responsiveness to change in the scholarly literature (e.g., De Meuse, 2019).

Certainly for leaders in contemporary organizations, adapting and adjusting to changing environments appear more important than ever: Leaders must continue to improve themselves in order to be effective (Calarco, 2020). Specifically, leaders need to be highly adaptable to new situations and challenges due to the rapidly changing global economy, uncertain business environments, and the unpredictability of organizational life in general (Norton, 2010). A recent example has been the response of leaders to the threats posed by the global COVID-19 pandemic in 2020–2022. Indeed, leaders who flexibly adapt to the new situation are crucial to the survival of contemporary organizations (Pasmore and Mallis, 2020), and this change-oriented behavior is at the core of leadership (Yukl, 2012). However, leaders differ in their ability in being flexible and adaptive to changing environments (Yukl and Mahsud, 2010). Consequently, a leader’s responsiveness to change (i.e., a leader’s learning agility) is key in the selection and development of future leaders.

Accordingly, learning agility is a popular concept in daily leadership practice (e.g., Dai et al., 2013; De Meuse, 2017). Indeed, a benchmarking study showed that practitioners frequently assess learning agility in their leadership selection and development programs (Church et al., 2015). However, as they usually “do not have publication as their top priority” (De Meuse et al., 2012), the number of academic studies on learning agility is limited (i.e., we found 40 studies in 26 years; most of these studies referred to commercial learning agility measures and their relation to leadership). So far, this emerging line of research has demonstrated the importance of learning agility for effective leadership by linking it to leader potential and performance (De Meuse, 2017).

Although scholars acknowledge that learning agility relates to leader effectiveness (e.g., De Meuse et al., 2010, 2012), they disagree on what learning agility comprises and, consequently, how to measure it (DeRue et al., 2012b; De Meuse, 2017). Indeed, prior studies have failed to clearly conceptualize learning agility *and* clarify its dimensions. Moreover, practitioners have developed different measures that are restricted by copyright protection (e.g., Lombardo and Eichinger, 2000; De Meuse et al., 2010; Hoff and Burke, 2018) and therefore inhibit the accumulation of scientific knowledge.

Hence, the objective of this study is to develop and validate a scale to measure the learning agility of (future) leaders, denoted the Leadership Learning Agility Scale (LLAS), with the aim to stimulate more academic research on this important topic. We contribute to the literature by (a) further conceptualizing the learning agility construct, (b) clarifying the underlying dimensions, and (c) developing a scale to measure it. Thus, we develop the LLAS following an approach in which theory development precedes measure development (Hinkin, 1998), and we provide initial evidence of its reliability and construct validity.

### Conceptualizing learning agility

#### Current definitions

Initially, learning agility was broadly conceptualized as “the willingness and ability to learn new competencies in order to perform under first-time, tough, or different conditions” (Lombardo and Eichinger, 2000). This definition served as a base for successive research (e.g., Dries et al., 2012; Dai et al., 2013), although it has been criticized for being too similar to a general ability to learn. Therefore, DeRue et al. (2012a) redefined learning agility as “the ability to come up to speed quickly in one’s understanding of a situation and move across ideas flexibly in service of learning both within and across experiences” (p. 262–263). Although this narrower definition has been criticized for being impractical (i.e., it fails to include motivational attributes that help to identify and develop high-potential employees; De Meuse et al., 2012) or for being too similar to general intelligence (e.g., Wang and Beier, 2012), it stimulated researchers to increase the conceptual clarity of learning agility (DeRue et al., 2012b). Moreover, different interpretations of the “agility” term sparked a scholarly discussion; some scholars related agility to its traditional meaning and thus focused on the speed and flexibility of learning (e.g., DeRue et al., 2012a; Hoff and Burke, 2018), whereas others related agility to being more behaviorally flexible in one’s social interaction (e.g., De Meuse et al., 2010). Because leadership is widely referred to as a process of social influence (Chemers, 2001) in which the quality and not the speed of social learning is key (De Meuse et al., 2012), it seems sensible not to include “speed of learning” when conceptualizing learning agility (De Meuse, 2017).

In current definitions, learning agility has often been described in terms of its outcomes (e.g., “to come up to speed quickly, to move across ideas flexibly” in DeRue et al., 2012a; “to perform successfully” in De Meuse, 2017), leading to a tautology. Conceptualizing a construct in terms of its outcomes is problematic both at a practical and theoretical level because it makes it impossible to distinguish learning agility as a predictor from learning agility outcomes (Suddaby, 2010). Thus, a conceptual refinement is needed.

#### Proposed definition

Four aspects are crucial when conceptualizing learning agility. First, in the context of leadership as a process of social influence (Chemers, 2001), learning agility refers to one’s aptitude to recognize and apply learnings regarding personal and relational aspects of social situations (Harris et al., 2022). In these situations, individuals emotionally and socially learn through human interaction. In contrast, learning agility does not focus on individual’s learning strategies or meta-cognitive processes (De Meuse et al., 2010, 2012). Second, learning agility is specifically linked to leader effectiveness (e.g., Dai et al., 2013; De Meuse, 2017), although one could argue that it can be a relevant trait for all employees (e.g., Gallardo-Gallardo et al., 2013). Nevertheless, leaders have a significant impact on both their subordinates’ and overall organizational performance (Hogan and Kaiser, 2005). Therefore, to enable strategic decisions on whom to select and develop as leaders (Collings and Mellahi, 2009), we focus on leadership learning agility. Third, current definitions include not only ability but also motivational components. Fourth, learning agility is linked to active learning and development (e.g., Lombardo and Eichinger, 2000; De Meuse et al., 2010), which implies that the drive, or the action to achieving something is more important than wanting to achieve something (TacSource, 2019). Hence, as our starting definition, we conceptualize leadership learning agility as the aptitude and willingness to learn from social experiences, and the drive to apply those lessons in new and challenging leadership roles.

### Underlying dimensions

Learning agility is a multidimensional construct (e.g., DeRue et al., 2012a; Dai et al., 2013), and – in line with current definitions which embody a reflective measurement approach – we posit that leadership learning agility represents a second-order construct (Wong et al., 2008), and that its dimensions represent first-order constructs (Edwards, 2001). In order to capture a broad leadership learning agility domain, we reviewed previously proposed learning agility dimensions using the following inclusion criteria: dimensions should (a) fit in our refined definition, (b) be distinguishable from other constructs such as personality, (c) not be described in terms of their desirable outcomes, and (d) comprise dimension labels that correspond with their respective definitions. Table 1 shows the posited leadership learning agility domain and the overlap and differences with the dimensions described in three most commonly used commercial learning agility scales. As shown in Table 1, our conceptualization of leadership learning agility differs from these commercial scales. We excluded results agility (i.e., described as individuals “who get results…”; Lombardo and Eichinger, 2000), because we consider “getting results” as an outcome. Moreover, we excluded mental agility due to the presumed overlap with other dimensions as illustrated below.

**Table 1:** Leadership learning agility domain compared with three commercial measures.

Present research	Consulting Firm, Country		
	Korn Ferry International, United States	Leader’s Gene Consulting, China	EASI Consult, Unites States
**(Leadership) Learning agility dimensions**			
LLAS	viaEDGE™	TALENT_x7_	Burke LAI™
Learning Through Social Interaction	People Agility	Interpersonal Acumen	Collaborating
–	–	–	Interpersonal Risk Taking
Developing Systematically	Change Agility	Change Alacrity	Experimenting
–	Results Agility	Drive to Excel	Performance Risk Taking
–	Mental Agility	Cognitive Perspective	Flexibility
Knowing Oneself	Self-Awareness	Self-Insight	Reflecting
Seeking Feedback	–	Feedback Responsiveness	Feedback Seeking
–	–	Environmental Mindfulness	–
–	–	–	Speed
–	–	–	Information Gathering
Developing Leadership			
**(Leadership) Learning agility conceptualizations**			
A. “The aptitude and willingness to learn from social experiences, and the drive to apply those lessons in new and challenging leadership roles”			
B. “The willingness and ability to learn new competencies in order to perform under first-time, tough, or different conditions” (Lombardo and Eichinger, 2000, p. 323)			
C. “The ability and willingness to learn quickly, and then apply those lessons to perform well in new and challenging leadership situations” (De Meuse, 2017, p. 272)			
D. “Dealing with new experiences flexibly and rapidly by trying new behavior, getting feedback on these attempts, and making quick adjustments so new learning will be realized when you do not know exactly what to do” (Hoff and Burke, 2018, p. 9)			

We extended the “learning agility factor comparisons among three assessments” from De Meuse (2017, p. 276).

#### Problems associated with current dimensions

Initially, Lombardo and Eichinger (2000) described five learning agility dimensions (Table 1), and the corresponding commercially available scale has been used in several studies (e.g., Dries et al., 2012; Dai et al., 2013). However, some scholars have argued that there are concerns related to this commonly used scale (e.g., a lack of careful theoretical consideration of the underlying dimensions), and that the field needs a new theoretically grounded and psychometrically sound learning agility measure (DeRue et al., 2012a,b).

Indeed, there are several conceptual and empirical issues concerning the conceptualizations of the initial dimensions. First, similar to current learning agility definitions, some dimensions have been described in terms of their outcomes. For instance, people agility was described as individuals “who treat others constructively …” (Lombardo and Eichinger, 2000). Second, academic research replicating the underlying dimensions is lacking (De Meuse, 2017), which impedes theory building and testing. Third, some dimensions seem to conceptually overlap with other predictor constructs such as personality or cognitive ability. To illustrate, mental agility was described as “people who think through problems from a fresh point of view [overlap with openness to experience] and are comfortable with complexity [overlap with cognitive ability] …” (Lombardo and Eichinger, 2000). Finally, some of the terms used to initially label and to subsequently conceive the different dimensions do not seem to fit their content. For example, the phrase “people who are cool and resilient under the pressures of change” [part of people agility] seems to overlap with the change agility label, which is conceived as “people who are curious, have a passion for ideas, like to experiment with test cases, and engage in skill-building activities” (Lombardo and Eichinger, 2000).

#### Probable leadership learning agility dimensions

**Figure 1:**
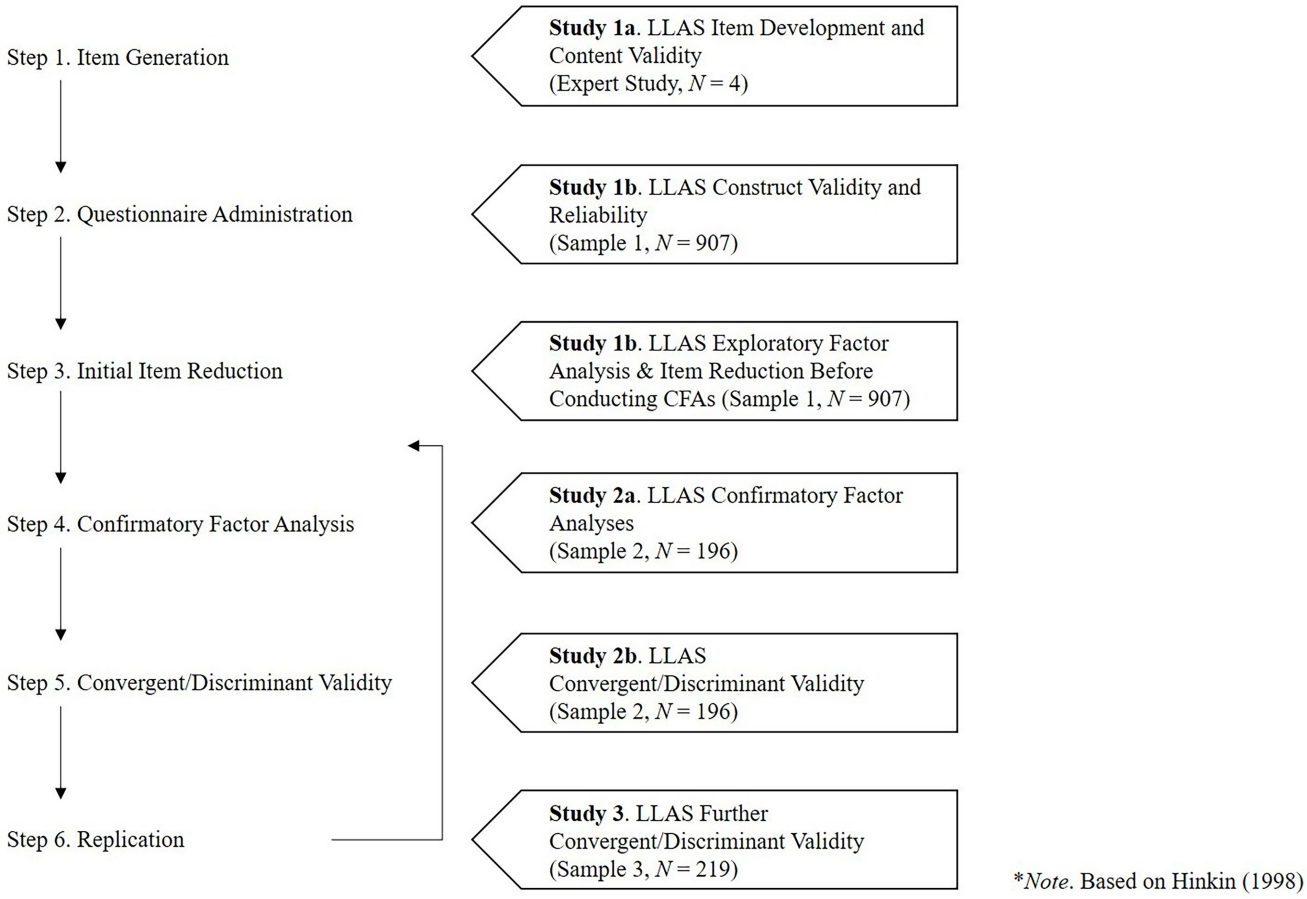
Six-step scale development approach* and overview of the different studies.

Building onto prior theory, we posit the following leadership learning agility dimensions. First, *Learning Through Social Interaction* refers to the extent to which individuals learn through social interaction (Reed et al., 2010), and seek novel social interactions to learn from. This dimension emphasizes the social components of learning (De Meuse et al., 2012). Next, *Developing Systematically* refers to the extent to which individuals seek opportunities to engage in (non-)formal learning activities in work environments. This dimension emphasizes the continued and deliberate practice of specific aptitudes, as a necessary condition for the growth of those aptitudes into future excellence (Nijs et al., 2014). Then, *Knowing Oneself* refers to the extent to which individuals are reflective and know themselves; recognizing their skills, strengths, weaknesses, blind spots, and hidden strengths (De Meuse, 2017). This dimension emphasizes the insights that individuals gain through internalization and reflection of those lessons that they learn from prior experience (Nesbit, 2012; De Meuse, 2017). Additionally, *Seeking Feedback* refers to the extent to which individuals solicit, listen to, and accept personal feedback from others, consider its merits, and subsequently take corrective action for performance improvement (De Meuse, 2017). This dimension emphasizes active feedback seeking from others in order to grow and develop (De Meuse et al., 2010). Finally, *Developing Leadership* refers to the extent to which individuals seek opportunities to engage and put effort in those developmental activities to develop oneself within a social context. This dimension emphasizes a drive and a preference to develop leadership skills in order to reach mutual organizational goals (Yukl, 2012). This drive is relevant for both leaders and for employees that take on informal leadership roles, because leadership is increasingly seen as a “mutual influence process independent of any formal role or hierarchical structure” (DeRue and Ashford, 2010). For instance, more and more organizations are implementing teams that work with self-managing agile practices (Junker et al., 2021), in which leadership is diffused among team members. Next, we developed a new scale to measure leadership learning agility (i.e., the LLAS) and examined its construct-related validity in three studies. We followed a six-step scale development process based on Hinkin (1998). Figure 1 provides an overview of the different studies,^1^ as well as the six-step approach toward the development of the LLAS.

## Study 1: Step 1–3, LLAS item development, factor structure, and reliability

In Study 1 we developed a scale to measure leadership learning agility that can be applied in a work-related context. Based on the literature review (i.e., 40 articles) and the dimension conceptualizations proposed above, we developed items for each of the posited leadership learning agility dimensions. Hence, we followed a deductive item development approach, as the theoretical foundation of prior studies provided enough information to generate an initial item set (Hinkin, 1998). The first and second author produced and (re)reviewed the initial item set in several rounds, guided by the specific dimension definitions.

Because prior research has shown that contextualized items increase criterion-related validity (e.g., Holtrop et al., 2014), we refer to specific workplace behavior in the items and item instructions. This contextualization of items is in contrast with prior learning agility scales, which contain more generic items (e.g., Lombardo and Eichinger, 2000). Although we decided to develop an entirely new item pool, we did examine the content of existing scales that might relate to specific dimensions such as Knowing Oneself (Grant et al., 2002) and Seeking Feedback (Tuckey et al., 2002) in order to ensure that we did not exclude relevant topics.

Concerning the measurement method, early learning agility measures used other reports as measurement approaches (e.g., Lombardo and Eichinger, 2000), whereas contemporary measures usually use self-assessments (e.g., Dai et al., 2013; Hoff and Burke, 2018). Although both self-reports and other reports could be relevant when assessing leadership learning agility, we developed a self-report scale because such a scale matches a self-directed learner attitude and self-observation (Nesbit, 2012), which relates to active learning and self-motivation.

### Study 1a: Step 1, LLAS item development and content validity (expert study)

Initially, we developed an item pool of 104 items with a 5-point Likert-type scale ranging from “1” (*strongly disagree*) to “5” (*strongly agree*). To assess the content validity of the leadership learning agility dimensions, its definitions, and corresponding items, we conducted an expert study in which we asked four subject matter experts to indicate on a 5-point Likert-type scale ranging from “1” (*strongly disagree*) to “5” (*strongly agree*) for each item how much this item represented its specific dimension. In addition, we asked feedback on the wording of the items *via* open-ended questions (i.e., “Please provide any suggestions or feedback regarding the wording of the items above”). The four subject matter experts, two of whom were female, were knowledgeable academics within the HRM and Industrial and Organizational Psychology field. Then, we calculated the averages and standard deviations per item. Items scoring on average “3” or lower and/or with a standard deviation higher than “1” were either rewritten or deleted. Additionally, based on the feedback we received via the open-ended questions, we adjusted specific item content (e.g., related to unclear terminology). After this expert study, our item pool was reduced to 89 items.

### Study 1b: Step 2–3, LLAS construct validity and reliability (sample 1)

#### Study 1b: Method

##### Study 1b: Step 2, procedure and participants

Next, we administered the LLAS to a sample of platform workers. With regard to the representativeness of our sample, we aimed for a sample that included both employees that did not yet have any (in)formal leadership experience, as well as participants that did have experience in leading others from (in)formal leadership roles. With this approach, we followed guidelines from the scale development literature, as Worthington and Whittaker (2006) stated that “it is not necessary to closely represent any clearly identified population as long as those who would score high and those who would score low are well represented [Gorsuch, 1997]” (p. 816). To determine our a priori sample size, we used a subject-to-item ratio of 10:1 (Costello and Osborne, 2005). We used Prolific Academic, an online platform known for its high-quality data, research naïve, and diverse participants (Peer et al., 2017). A total of 941 participants started our questionnaire. We asked all participants to indicate their (a) informed consent, (b) gender, (c) age, (d) years of work experience, (e) work hours per week, (f) highest educational attainment, and (g) managerial experience. We added three control questions to check participants’ attention span (e.g., “This is an attentiveness check; please indicate *neutral*”). We removed eight participants from our dataset based on failed attention checks, and 26 participants were timed-out by Prolific Academic after 30 min test time. The total test time was ~10 min. We paid the respondents £5.00 per hour.

In total, 907 participants took part in Study 1b. Roughly half of the respondents (56%) reported US/UK English as their first language, and nearly all others (37%) reported different European languages as their first language. Roughly half of the sample was male (57%). The average age of the participants was 30.7 (*SD* = 9.7) years. The educational level of the participants was relatively high: 63% held at least a college or university degree and an additional 12% was currently in college or university. On average, participants worked 34.3 (*SD* = 10.7) h per week, and had 10.1 (*SD* = 9.3) years of work experience, and 2.7 (*SD* = 4.6) years of managerial experience. Roughly half of the respondents (59%) had at least 1 year of managerial experience.

##### Study 1b: Measures

*Leadership learning agility* was measured with the LLAS (89 items) developed in Study 1a.

#### Study 1b: Results

##### Study 1b: Step 3, exploratory factor analysis

We appraised the underlying factor structure by conducting an exploratory factor analysis (EFA). We ran a maximum likelihood factor analysis (FA) in SPSS v.26 to understand the latent factors that account for the shared variance among items (Worthington and Whittaker, 2006). We used a maximum likelihood method, as this is the preferred method if the data are generally normally distributed (Costello and Osborne, 2005). Accordingly, as an initial step before conducting any analyses, we checked the normal probability plots, as well as the absolute skewness and kurtosis values (these absolute values should be smaller than 2 and 7, respectively for sample sizes >300; Kim, 2013; Demir, 2022) of all 89 LLAS items. These initial analyses showed that the data were generally normally distributed. Moreover, we used an oblique rotation method (i.e., direct oblimin) because we expected the underlying dimensions to be correlated (Costello and Osborne, 2005). The Kaiser-Meyer-Olkin (KMO) measure of sampling adequacy was 0.96, so we could proceed. To reduce the risk of factor over-extraction, we used the scree plot procedure rather than eigenvalues, which indicated four factors to retain (Worthington and Whittaker, 2006). Subsequently, to obtain the most interpretable factor solution (Costello and Osborne, 2005), we ran multiple FAs with three to six factors and compared the item pattern matrices.

In all models, the reverse-coded items loaded on a separate factor, indicating a reverse scoring method effect rather than a conceptually meaningful factor (Magazine et al., 1996). Hence, we decided to delete these items from subsequent analyses. Of the different factor models, the three-factor model contained the cleanest factor structure (i.e., including item loadings above 0.30, no item cross-loadings, no factors with fewer than three items, and factors with the largest number of items per factor; Costello and Osborne, 2005; Worthington and Whittaker, 2006), and thus initially showed the best fit to the data. Finally, we examined the items within each factor to assess the extent to which the items had a common meaningful core related to our a priori dimensions (Worthington and Whittaker, 2006). The items reflecting Developing Leadership, Seeking Feedback, and Developing Systematically loaded on separate factors in the three-, four-, and five-factor solutions.

##### Study 1b: Item reduction before conducting confirmatory factor analyses

Then, to set the fundament for subsequent confirmatory factor analyses (CFAs), we reevaluated all EFA factor solutions and corresponding items (Schreiber et al., 2006). We used three guidelines to decide upfront which factors and items to retain in the CFA models (i.e., to test later, in a new sample): (a) solid factors contain at least five items with item loadings of 0.50 or higher (Costello and Osborne, 2005), (b) a parsimonious set of items per factor is preferable (Hinkin, 1998), thus we deleted those items that were too similar in content, and (c) item categorization is based on the statistical results rather than on our preconceived categorization of items per factor (i.e., to prevent researchers’ bias; Worthington and Whittaker, 2006). Additionally, in a subsequent CFA study, we aimed for a sample size of ~200 respondents. Accordingly, to ensure a subject-to-item ratio of 10:1, we used two final guidelines to decide upfront which factors and items to retain in the CFA models: (d) a similar number of items per factor within a model and (e) not too many, nor too few items per model. To ensure that the factor solutions did not change after deleting items, we conducted a final EFA (i.e., no a priori factors extracted) which showed similar factorial results. Overall, the Developing Leadership, Seeking Feedback, and Developing Systematically factors seemed most supportable. In the three-factor solution, these factors were measured with 18 items and explained ~56% of the shared variance in the data. The coefficient alphas of the (sub)scales ranged from 0.81 to 0.89. These high alphas are fundamental in the phase of scale development (DeVellis, 2017). Table 2 shows the items in the three-factor solution and their means, standard deviations, internal consistencies, and factor loadings. The intercorrelations of the three factors ranged from *r* = 0.36 to *r* = 0.48, all *p*’s < 0.01 (Table 3, Sample 1).

**Table 2:** LLAS items, means, standard deviations, internal consistencies, and factor loadings (sample 1; *N* = 907).

Nr.	Item	Factor					
		*M*	SD	*α*	1	2	3
	**LLAS Total**			0.89			
	*Factor 1: Developing leadership*			0.87			
20	At work, I put effort in trying to develop contrasting influential styles (e.g., taking the lead and empowering others)	3.50	0.96		0.66		
46	I put effort in getting better in influencing others to reach our project goals	3.60	0.96		0.75		
47	I reflect on how to effectively influence my colleagues in our social interactions	3.46	0.97		0.79		
57	I try to influence the development of my co-workers to attain our project goals	3.51	0.94		0.71		
67	I focus on how to effectively lead my peers toward our team goals at work	3.60	0.90		0.65		
87	I focus on how to become an influencer in my organization to reach our targets	3.17	1.06		0.70		
	*Factor 2: Seeking feedback*			0.81			
18	At work, I carefully evaluate the feedback I receive from others to learn from it	3.98	0.78			0.60	
29	At work, I conceive feedback as a fundamental tool to my performance improvement	3.97	0.82			0.58	
34	I act upon the feedback I receive from peers to improve my job performance	3.88	0.73			0.67	
44	I examine patterns in my own behavior based on the feedback I receive from co-workers	3.83	0.79			0.60	
60	I take action when a colleague gives feedback to improve my performance	3.94	0.74			0.65	
75	I adjust my behavior based on the feedback I receive from colleagues	3.73	0.82			0.64	
	*Factor 3: Developing systematically*			0.82			
04	At work, I participate in learning activities (e.g., trainings and workshops) to personally develop	3.92	0.94				0.76
10	I take part in developmental activities to improve my task-and relational skills at work	3.91	0.82				0.58
35	I self-initiate learning activities to improve my job performance	3.80	0.90				0.51
56	I participate in trainings because I want to continue developing at work	3.95	0.85				0.66
61	I take part in educational programs besides my working activities	3.64	1.06				0.58
82	At work, I participate in educational opportunities to further develop	3.89	0.87				0.77

LLAS, Leadership learning agility scale.

**Table 3:** Means, standard deviations, correlations, and internal consistencies of the study variables in sample 1 (*N* = 907) and 2 (*N* = 196).

	Sample		*M*	SD	1	2	3	4	5	6	7	8	9	10	11	12	13	14
1	Age	1	30.74	9.70	-													
		2	30.23	10.57	-													
2	Gender	1	0.43	0.49	0.06	-												
		2	0.45	0.50	0.05	-												
3	Educational level	1	4.71	1.74	0.26**	0.08*	-											
		2	4.42	1.74	0.11	0.11	-											
4	Work hours	1	34.26	10.69	0.22**	−0.18**	0.23**	-										
		2	21.89	17.66	0.07	0.08	0.26**	-										
5	Work experience	1	10.11	9.33	0.87**	0.09**	0.10**	0.14**	-									
		6	Managerial experience	1	2.74	4.64	0.59**	−0.002	0.15**	0.21**	0.60**	-								
2	2.40			4.96	0.57**	0.04	0.11	0.05	0.10	-								
7	LLAS Total	1	3.74	0.52	0.02	0.07*	0.14**	0.11**	−0.01	0.13**	(0**.89**)							
		2	3.55	0.55	0.13	0.13	0.17*	0.15*	0.11	0.29**	(0**.89**)							
8	Developing Leadership	1	3.47	0.75	0.02	−0.04	0.06	0.14**	0.004	0.21**	0.81**	(0**.87**)						
		2	3.24	0.82	0.11	0.12	0.09	0.10	0.15*	0.31**	0.82**	(0**.89**)						
9	Seeking Feedback	1	3.88	0.56	−0.04	0.08*	0.09**	0.03	−0.05	0.004	0.73**	0.36**	(0**.81**)					
		2	3.80	0.51	0.07	0.11	0.13	0.01	−0.04	0.15*	0.69**	0.33**	(0**.74**)					
10	Developing Systematically	1	3.85	0.66	0.06	0.15**	0.18**	0.08*	0.01	0.07*	0.83**	0.48**	0.48**	(0**.82**)				
		2	3.63	0.74	0.14	0.07	0.18*	0.20**	0.12	0.20**	0.84**	0.49**	0.47**	(0**.85**)				
11	ICAR	2	7.12	3.32	−0.17*	−0.18*	0.20**	0.12	−0.08	−0.14	−0.12	−0.22**	0.06	−0.07	**(0.74)**			
12	AMM Total	2	3.56	0.57	0.17*	0.16*	0.14	0.13	0.01	0.17*	0.60**	0.51**	0.45**	0.46**	−0.14	**(0.79)**		
13	Achievement Thou	2	3.79	0.62	0.17*	0.17*	0.12	0.08	−0.02	0.12	0.54**	0.42**	0.48**	0.41**	−0.09	0.93**	**(0.79)**	
14	Achievement Beha	2	3.04	0.75	0.11	0.07	0.11	0.17*	0.06	0.19**	0.47**	0.48**	0.22**	0.37**	−0.16*	0.74**	0.44**	**(0.56)**

Internal consistencies are presented in bold and between parentheses. Gender is coded as 0 = male, 1 = female. Educational level is coded as 1 = <12 years, 2 = high school graduate, 3 = currently in college/university, 4 = some college/university, but did not graduate, 5 = college/university degree, 6 = currently in graduate/professional school, 7 = graduate/professional school degree, and 8 = PhD. Work hours per week. Work experience (Sample 1) in years. Managerial experience in years. LLAS, Leadership learning agility scale; ICAR, International cognitive ability resource; AMM, Achievement motivation measure. The ICAR M and SD refer to the ICAR sum score. Achievement Thou = Achievement thoughts, Achievement Beha = Achievement behaviors. Note that the analyses for the variable gender are based on *N* = 900 (Sample 1) and *N* = 195 (Sample 2), as seven (Sample 1) and one (Sample 2) participants indicated “other” as gender value.

*p < 0.05, **p < 0.01; two-tailed tests.

#### Study 1b: Discussion

In Study 1b, we showed that leadership learning agility comprises a Developing Leadership, Seeking Feedback, and Developing Systematically dimension. In contrast, Learning Through Social Interaction and Knowing Oneself failed to clearly load onto separate factors; only one of these dimension items was retained and recategorized in Developing Leadership. Thus, the empirical results showed that the motivational (i.e., and not the aptitude) component in our construct definition was most important. Based on these results, we decided to adjust our starting definition of leadership learning agility by removing the “aptitude” term from it. Next, we validated the findings of Study 1b in a new research sample.

## Study 2: Step 4–6, CFAs, convergent, and discriminant validity (sample 2)

### Study 2: Method

#### Study 2: Procedure and participants

Again, we used Prolific Academic to collect our data among a sample of platform workers, representing both employees that did not yet have any (in)formal leadership experience, as well as participants that did have experience in leading others from (in)formal leadership roles (i.e., similar to Study 1b). A total of 220 participants started our questionnaire containing the 89 LLAS items from Study 1b, and two additional measures (described below, under Study 2b). We asked all participants to indicate their (a) informed consent, (b) gender, (c) age, (d) work hours per week, (e) highest educational attainment, and (f) managerial experience. We removed five participants from our dataset based on failed attention checks, 15 participants stopped before finishing the full questionnaire, and we excluded four cases due to a missing completion code in Prolific Academic. The total test time in Study 2 was ~30 min. We paid the respondents £6.00 per hour.

In total, 196 participants took part in Study 2. Less than half of the respondents (40%) reported US/UK English as their first language, and nearly all others (55%) reported different European languages as their first language. Although the two Prolific research samples are roughly comparable based on this information, the sample in Study 2 included 16% more participants that reported different European languages (i.e., not US/UK English) as their first language. Roughly half of the sample was male (55%). The average age of the participants was 30.2 (*SD* = 10.6) years. The educational level of the participants was relatively high: 55% held at least a college or university degree and an additional 18% was currently in college or university. On average, participants worked 21.9 (*SD* = 17.7) h per week, and had 2.4 (*SD* = 5.0) years of managerial experience. Roughly half of the respondents (49%) had at least 1 year of managerial experience.

### Study 2a: Step 4, CFAs

#### Study 2a: Measures

*Leadership learning agility.* Leadership learning agility was measured with the LLAS (89 items) developed in Study 1a. We used the 89-item LLAS in Study 2a, to ensure that we could test all prior EFA models *via* a CFA procedure in a new sample. Hence, the analyses in Study 2a are based on the 89-item version of the LLAS.

#### Study 2a: Confirmatory factor analyses

We conducted CFAs (AMOS v.26) and used several indices with stringent cutoff values to assess model fit (Hu and Bentler, 1999; Schreiber et al., 2006; Worthington and Whittaker, 2006): (1) the Chi-square value (*x*^2^) with the number of degrees of freedom (df); (2) the standardized root mean squared residual (SRMR; value ≤0.08), (3) the root mean square of approximation (RMSEA; value ≤0.06) with its 90% confidence interval and its *p* of close fit (Pclose; value >0.05); (4) the Akaike information criterion (AIC; the smaller value the better); (5) the Tucker–Lewis index (TLI; value ≥0.95); and (6) the comparative fit index (CFI; value ≥0.95).

### Study 2a: Results

**Figure 2:**
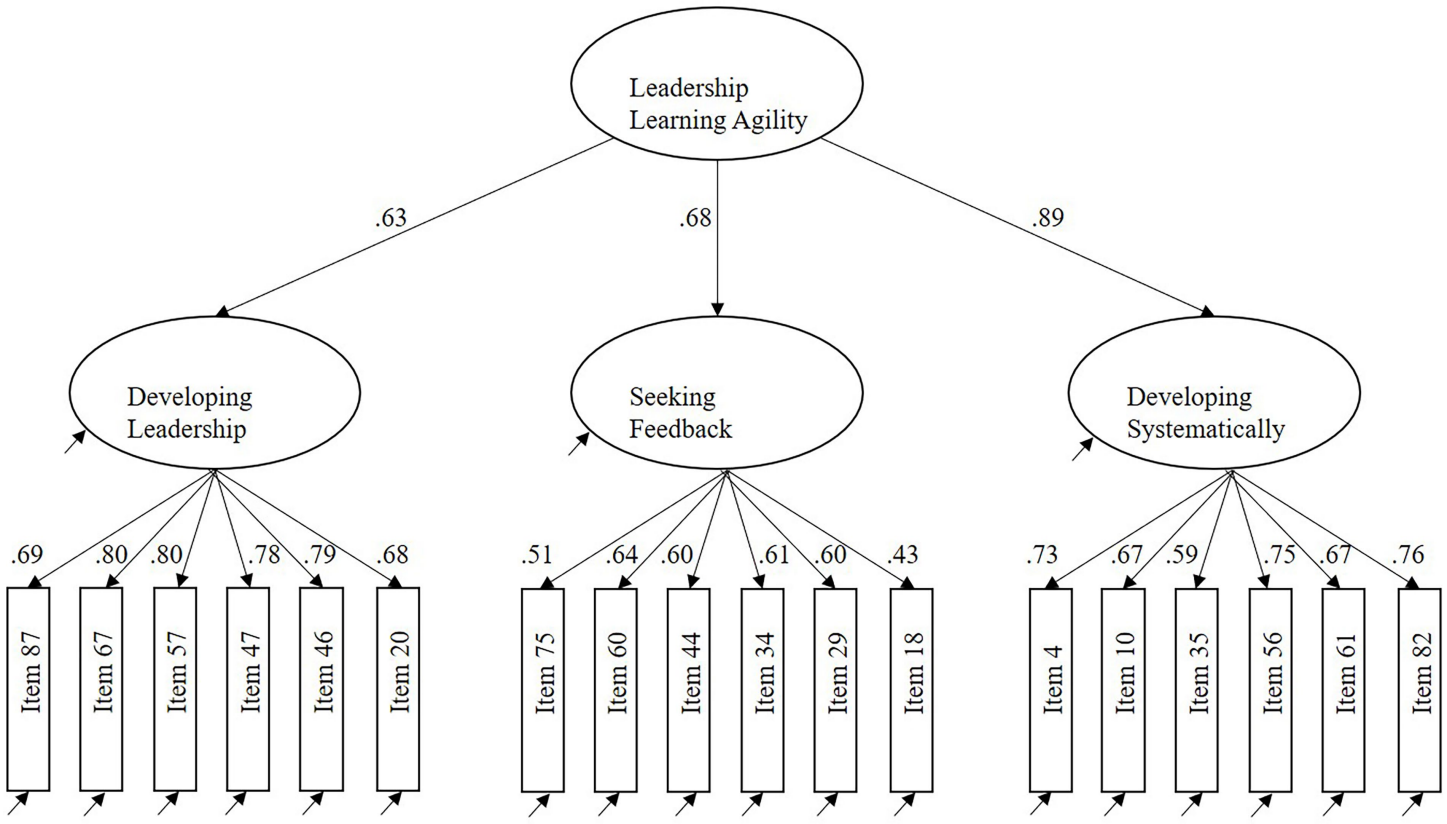
The three-factor model and its factor loadings (sample 2, *N* = 196).

Table 4 shows the results of the different CFA models (i.e., the EFA models after item reduction, as described in Study 1b). Similar to the EFA results, the three-factor model showed the best fit to the data, with TLI and CFI values of 0.95; a SRMR value of 0.051; a RMSEA value of 0.049 [90% CI = 0.0.33–0.063, Pclose = 0.543]; and an AIC value of 271.07. We did *not* use any CFA post-hoc modifications (Schreiber et al., 2006). As an extra check, we tested the fit of a one-factor model with the items of the three-factor model, this one-factor model showed inferior results (Table 4). In the three-factor model (Figure 2), all items significantly loaded on their proposed first-order factors (item loadings ranged from 0.43 to 0.80, all *p*’s < 0.001), which all significantly loaded on the second-order factor (loadings ranged from 0.63 to 0.89, all *p*’s < 0.001).

**Table 4:** Goodness-of-fit indices of the confirmatory factor analyses of the leadership learning agility scale (sample 2; *N* = 196).

Model (# factors)	*χ* ^2^	df	TLI	SRMR	CFI	RMSEA [90% CI]	Pclose	AIC
One	552.94	135****	0.65	0.108	0.69	0.126 [0.115–0.137]	0.000	624.94
Two	272.41	134****	0.90	0.068	0.92	0.073 [0.060–0.085]	0.002	346.41
Three/correlated model	193.07	132****	0.95	0.051	0.95	0.049 [0.033–0.063]	0.543	271.07
Three/hierarchical model	193.07	132****	0.95	0.051	0.95	0.049 [0.033–0.063]	0.543	271.07
Four	221.24	131****	0.92	0.066	0.93	0.059 [0.046–0.073]	0.125	301.24
Five	529.70	270****	0.83	0.081	0.84	0.070 [0.061–0.079]	0.000	639.70

*χ*^2^, Chi-square with df, degrees of freedom; TLI, Tucker Lewis index; SRMR, standardized root mean squared residual; CFI, comparative fit index; RMSEA [90% CI], root mean square of approximation [90 percent confidence interval]; Pclose, p of close fit; AIC, Akaike information criterion.

**** *p* < 0.0001.

The fit indices of a hierarchical three-factor model are identical to those of a more parsimonious correlated three-factor model. Hence, to demonstrate that leadership learning agility is a higher-order construct (Crede and Harms, 2015), we examined the correlations between its dimensions. The correlation between Developing Leadership and Seeking Feedback was 0.33, Developing Leadership and Developing Systematically was 0.49, and Seeking Feedback and Developing Systematically was 0.47 (all *p*’s < 0.01). These correlations indicate that a higher-order construct is plausible (Crede et al., 2017). The coefficient alphas of the (sub)scales ranged from 0.74 to 0.89 (Table 3, Sample 2).

### Study 2a: Discussion

Study 2a confirmed the factor structure of Study 1b in a new sample. We found a good fit for the LLAS three-factor solution. The final version of the LLAS comprises 18 items (Supplemental Table S1).

### Study 2b: Step 5, convergent and discriminant validity

#### Study 2b: Hypotheses and research question

Generally, learning agility (i.e., the overall construct) is linked to leader potential, performance, and success (e.g., Dai et al., 2013; De Meuse, 2017). Hence, we expect that leadership learning agility is related to achievement motivation because this latter construct relates to high performing individuals (McClelland, 1985). Achievement motivation is conceived as “the persistent impulse to attain a high standard of performance” (Smith et al., 2020) and includes both thoughts and behaviors. Thus, we expect that leadership learning agility is positively related to achievement thoughts (*Hypothesis 1a*). Likewise, we expect that leadership learning agility is positively related to achievement behaviors (*Hypothesis 1b*). Additionally, as the Developing Systematically items specifically focus on one’s effort to initiate self-developmental activities, we expect that this dimension is more strongly related to achievement thoughts than Developing Leadership and Seeking Feedback (*Hypothesis 2a*). Likewise, we expect that Developing Systematically is more strongly related to achievement behaviors than Developing Leadership and Seeking Feedback (*Hypothesis 2b*).

On-the-job learning and adaptability have been positively related to cognitive ability in prior studies (e.g., Schmidt and Hunter, 2004; Stasielowicz, 2020). However, leadership learning agility, as measured by its three dimensions in the current study, refers to learning from social situations (De Meuse et al., 2012; DeRue et al., 2012a), rather than for instance finding correct and quick solutions to cognitive problems, as measured by cognitive ability (e.g., Schmidt and Hunter, 2004). Indeed, previously, scholars have suggested that learning agility is unrelated to cognitive ability (e.g., Eichinger and Lombardo, 2004); however, research that specifically examined this relationship is scarce. Therefore, we decided to exploratively examine the relationship between leadership learning agility and cognitive ability by answering the following research question: “To what extent is leadership learning agility correlated with cognitive ability?”

#### Study 2b: Measures

*Achievement motivation.* Achievement motivation was measured with the Achievement Motivation Measure (AMM; Smith et al., 2020). An example item of achievement thoughts is “I can keep my mind on a task for a long period of time.” An example item of achievement behaviors is “I try and follow the rule: Business before pleasure.” The AMM uses a 5-point frequency scale ranging from “1” (*never*) to “5” (*always*). The coefficient alphas of the (sub)scales were 0.56 and 0.79 (Table 3), which is similar to values in prior research (Smith et al., 2020; CFA Sample).

*Cognitive ability.* Cognitive ability was measured with the International Cognitive Ability Resource (ICAR), measuring letter and number series, matrix reasoning, three-dimensional rotation, and verbal reasoning. The ICAR has been developed for use in an online research context. Coefficient alpha for the ICAR was 0.74, a slightly lower value compared with prior research (Condon and Revelle, 2014).

### Study 2b: Results

Table 3 (Sample 2) displays the means, standard deviations, internal consistencies, and correlations of all study variables. Confirming Hypothesis 1, the mean score of the LLAS was positively correlated with achievement thoughts (*r* = 0.54, *p* < 0.01; Hypothesis 1a) and behaviors (*r* = 0.47, *p* < 0.01; Hypothesis 1b). All LLAS dimensions were positively correlated with achievement thoughts and behaviors (Developing Leadership values were *r* = 0.42, *r* = 0.48; Seeking Feedback values were *r* = 0.48, *r* = 0.22; Developing Systematically values were *r* = 0.41, *r* = 0.37, respectively, all *p*’s < 0.01). We used Steiger’s *z*-test to compare these correlation coefficients (Lee and Preacher, 2013). The correlation coefficients of Developing Leadership (i.e., *z* = 3.47, *p* < 0.001, two-tailed) and Developing Systematically (i.e., *z* = −2.16, *p* = 0.03, two-tailed) with achievement *behaviors* were significantly stronger than the correlation coefficient of Seeking Feedback with achievement *behaviors*. All other differences in correlation coefficients were non-significant. Hence, Hypothesis 2a was not supported; we expected the correlations of achievement thoughts with Developing Systematically to be stronger than the correlations with the other LLAS dimensions. Moreover, Hypothesis 2b was partly supported, as the correlation between Developing Systematically (i.e., *r* = 0.37, *p* < 0.01) and achievement *behaviors* was significantly stronger than the correlation between Seeking Feedback (i.e., *r* = 0.22, *p* < 0.01) and achievement *behaviors*. However, the correlation between Developing Leadership (i.e., *r* = 0.48, *p* < 0.01) and achievement *behaviors* was also significantly stronger than the relationship between Seeking Feedback (i.e., *r* = 0.22, *p* < 0.01) and achievement *behaviors* (we expected the correlations of achievement behaviors and Developing Systematically to be significantly stronger than the correlations with the other LLAS dimensions).

#### Exploratory correlation analyses

The mean score of the LLAS was unrelated to the sum score of the ICAR (*r* = −0.12, *p* = 0.08). With regard to the LLAS dimensions, Developing Systematically (*r* = −0.07, *p* = 0.31) and Seeking Feedback (*r* = 0.06, *p* = 0.43) were unrelated to the sum score of the ICAR. However, Developing Leadership was negatively correlated with the sum score of the ICAR (*r* = −0.22, *p* < 0.01).

### Study 2b: Discussion

Study 2b showed that the LLAS was positively related to a motivational measure, showing adequate convergent validity. In contrast with Hypothesis 2, Developing Systematically was not more strongly related to achievement motivation than Developing Leadership and Seeking Feedback. Apparently, all LLAS dimensions comprise a common set of motives that drive individuals with high achievement motivation (Smith et al., 2020).

Additionally, the results of our exploratory analyses showed that at an aggregate level, the LLAS was unrelated to cognitive ability. In contrast, the Developing Leadership dimension was negatively correlated with cognitive ability. Recent meta-analytic results showed that the effects of ability and motivation (on performance) are compensatory rather than non-compensatory (Van Iddekinge et al., 2018). Thus, participants with a lower level of cognitive ability may feel a need to compensate their lower cognitive ability by seeking more opportunities to develop oneself.

## Study 3: Step 6, LLAS further convergent and discriminant validity (sample 3)

### Study 3: Method

#### Study 3: Procedure and participants

Next, we collected data within our professional network to administer the LLAS among a field sample of leaders, in order to replicate the scale development results of Studies 1 and 2. We aimed for a minimum sample size of 193 participants, based on an a priori power analysis (i.e., *r* = 0.20, 80% power, *α* = 0.05, two-tailed) in G*Power (Faul et al., 2009). We personally spoke to 121 contacts and subsequently sent them an email including a brief description of our research goal and a link to our questionnaire. We also asked them to forward this email to colleagues. In total, 320 participants started our questionnaire. We asked all participants to indicate their (a) informed consent, (b) gender, (c) age, (d) work hours per week, (e) highest educational attainment, (f) managerial experience, (g) the number of subordinates/peers currently supervised, (h) work field, and (i) country of residence. We included a personality and cognitive ability measure to further examine the construct validity of the LLAS. One hundred and one participants stopped before finishing the questionnaire. We did not have to remove any participants from our dataset based on failed attention checks. The total test time in Study 3 was ~30 min.

In total, 219 participants took part in Study 3. The majority of respondents (85%) reported a European country as their country of residence, with (80%) living in Netherlands. Roughly half of the sample was male (57%). The average age of the participants was 43.7 (*SD* = 10.5) years. The educational level of the participants was relatively high: 89% held at least a college or university degree. On average, participants worked 40.2 (*SD* = 10.3) h per week, and had 21.3 (*SD* = 10.9) years of work experience. The majority of respondents (83%) supervised at least one subordinate. Participants mainly worked in the consulting (26%), finance (22%), non-profit (18%), or information technology (7%) fields. Thus, compared with the participants in Study 1b and Study 2, this research sample included more leaders, who were older and higher educated.

### Study 3: Hypotheses

Although early scholars stated that learning agility is unrelated to personality (Eichinger and Lombardo, 2004), recently it was hypothesized that learning agility (i.e., the overall construct) most likely is related to various personality traits (De Meuse, 2017). The HEXACO model is a six-dimension structure of personality, which is replicated in lexical studies in multiple non-English languages (Ashton and Lee, 2007). The dimensions are: honesty-humility (H), emotionality (E), extraversion (X), agreeableness (A), conscientiousness (C), and openness to experience (O). As learning agility refers to the willingness and drive to learn in a social context, we expect a positive relationship of leadership learning agility with (a) extraversion, because interaction with others is key when focusing on developing leadership-relevant skills and when seeking feedback from others; (b) conscientiousness, because the accomplishment of work tasks is most probably linked to the continued and deliberate practice of specific aptitudes; and (c) openness to experience because, in general, having positive attitudes toward learning experiences seems important (Ashton and Lee, 2007). Thus, we expect that leadership learning agility is positively related to extraversion (*Hypothesis 3a*). Likewise, we expect that leadership learning agility is positively related to conscientiousness (*Hypothesis 3b*). Similarly, we expect that leadership learning agility is positively related to openness to experience (*Hypothesis 3c*). In addition, similar to our reasoning in Study 2b, we included cognitive ability to examine its relationship with leadership learning agility.

#### Study 3: Measures

*Leadership learning agility.* Leadership learning agility was measured with the LLAS (18 items) developed in Study 2a. The coefficient alphas for the (sub)scales ranged from 0.75 to 0.87 (Table 5). Again, we found a good model fit (TLI = 0.96, CFI = 0.96, SRMR = 0.064, RMSEA = 0.040 [90% CI = 0.023–0.054; Pclose = 0.866], AIC = 255.06). All items significantly loaded on their proposed first-order factors (item loadings ranged from 0.47 to 0.84, all *p*’s < 0.001), which all significantly loaded on the second-order factor (loadings ranged from 0.40 to 0.92, all *p*’s < 0.001; Heywood case corrected, because, despite the good model fit, the restricted standardized loading of Seeking Feedback was 1.06; Dillon et al., 1987). The correlations between the first-order factors ranged from 0.18 to 0.38 (all *p*’s < 0.01).

**Table 5:** Means, standard deviations, internal consistencies, and correlations of the study variables in sample 3 (*N* = 219).

	*M*	SD	1	2	3	4	5	6	7	8	9	10	11	12	13	14	15	16	17
1. Age	43.68	10.52	-																
2. Gender	0.43	0.50	−0.30**	-															
3. Edu. level	5.49	1.39	−0.08	0.08	-														
4. Work hours	40.22	10.28	0.004	−0.19**	−0.08	-													
5. Work experience	21.32	10.92	0.93**	−0.26**	−0.14*	0.01	-												
6. Subordinates no.	15.03	42.01	0.21**	−0.15*	−0.16*	0.13	0.19**	-											
7. LLAS Total	3.99	0.41	0.02	0.13	0.10	−0.01	0.04	−0.06	**(0.84)**										
8. Developing LS	3.90	0.56	0.25**	−0.19**	−0.05	0.10	0.24**	0.10	0.67**	**(0.80)**									
9. Seeking Feedback	4.14	0.42	−0.11	0.14*	0.04	−0.04	−0.10	−0.05	0.72**	0.38**	**(0.75)**								
10. Developing Syst	3.95	0.71	−0.10	0.28**	0.18**	−0.08	−0.07	−0.15*	0.78**	0.18**	0.37**	**(0.87)**							
11. ICAR	7.55	2.89	−0.26**	−0.02	0.08	−0.04	−0.30**	−0.16*	−0.10	−0.18**	0.02	−0.04	**(0.66)**						
12. H	3.75	0.52	0.11	0.10	−0.12	−0.05	0.09	0.05	0.10	0.04	0.08	0.10	0.01	**(0.70)**					
13. E	2.92	0.58	−0.21**	0.43**	0.10	−0.14*	−0.23**	−0.25**	0.05	−0.14*	0.08	0.16*	0.01	−0.07	**(0.77)**				
14. X	3.72	0.46	0.03	−0.08	0.02	0.02	0.04	0.11	0.32**	0.34**	0.20**	0.18**	−0.05	0.02	−0.24**	**(0.72)**			
15. A	3.05	0.51	0.03	0.08	−0.01	−0.10	0.02	−0.001	0.13	−0.04	0.15*	0.16*	−0.03	0.25**	−0.18**	0.09	**(0.71)**		
16. C	3.65	0.59	−0.11	0.04	0.003	0.07	−0.10	0.15*	0.25**	0.20**	0.22**	0.15*	−0.03	0.27**	−0.04	0.19**	0.04	**(0.80)**	
17. O	3.67	0.55	0.01	−0.03	0.09	0.11	−0.01	0.03	0.10	0.09	0.07	0.06	−0.05	−0.02	−0.04	0.05	−0.11	0.07	**(0.74)**

Internal consistencies are presented in bold and between parentheses. Gender is coded as 0 = male, 1 = female. Edu. level = Educational level, coded as 1 = <12 years, 2 = high school graduate, 3 = currently in college/university, 4 = some college/university, but did not graduate, 5 = college/university degree, 6 = currently in graduate/professional school, 7 = graduate/professional school degree, and 8 = PhD. Work hours per week. Work experience in years. LLAS = Leadership Learning Agility Scale. Developing LS = Developing Leadership. Developing Syst = Developing Systematically. ICAR = International cognitive ability resource. The ICAR M and SD refer to the ICAR sum score. The ICAR analyses are based on *N* = 215. H, Honesty-humility; E, Emotionality; X, eXtraversion; A, Agreeableness; C, Conscientiousness; O, Openness to experience.

*p < 0.05; **p < 0.01; two-tailed tests.

##### Personality

Personality was measured with the HEXACO–60 (Ashton and Lee, 2009) which uses a 5-point Likert-type scale ranging from “1” (*strongly disagree*) to “5” (*strongly agree*). The coefficient alphas of the subscales ranged from 0.70 to 0.80 (Table 5), which is similar to values in prior research (Ashton and Lee, 2009).

##### Cognitive ability

Cognitive ability was measured with the ICAR, also used in Study 2b. In order to control the total test time (Condon and Revelle, 2014), we imposed a time limit of 1 min per item. Participants saw a clock counting down and the next item would appear on the screen automatically. Based on participants’ feedback and sequentially missing value patterns for the Matrix Reasoning items, we deleted the scores of four participants. Hence, all ICAR analyses are based on a sample size of 215 participants. Coefficient alpha of the ICAR was 0.66. This value is lower compared with values in prior research (Condon and Revelle, 2014).

### Study 3: Results

Table 5 displays the means, standard deviations, internal consistencies, and correlations of all study variables. Partially in line with Hypothesis 3, the mean score of the LLAS was positively correlated with extraversion (*r* = 0.32, *p* < 0.01; Hypothesis 3a) and conscientiousness (*r* = 0.25, *p* < 0.01; Hypothesis 3b), but unrelated to openness to experience (*r* = 0.10, *p* = 0.14; Hypothesis 3c). Regarding the LLAS dimensions, extraversion (X) and conscientiousness (C) were positively correlated with Developing Leadership (X: *r* = 0.34; C: *r* = 0.20, both *p*’s < 0.01), Seeking Feedback (X: *r* = 0.20; C: *r* = 0.22, both *p*’s < 0.01), and Developing Systematically (X: *r* = 0.18, *p* < 0.01; C: *r* = 0.15, *p* = 0.02). However, openness to experience (O) was unrelated to Developing Leadership (*r* = 0.09, *p* = 0.18), Seeking Feedback (*r* = 0.07, *p* = 0.33), and Developing Systematically (*r* = 0.06, *p* = 0.35). Furthermore, the mean score of the LLAS was unrelated to emotionality (*r* = 0.05, *p* = 0.43), honesty-humility (*r* = 0.10, *p* = 0.15), and agreeableness (*r* = 0.13, *p* = 0.06). Regarding the LLAS dimensions and any significant relationships with specific personality dimensions, emotionality was negatively correlated with Developing Leadership (*r* = −0.14, *p* = 0.03), but positively correlated with Developing Systematically (*r* = 0.16, *p* = 0.02). Moreover, agreeableness was positively correlated with Seeking Feedback (*r* = 0.15, *p* = 0.02) and Developing Systematically (*r* = 0.16, *p* = 0.02).

#### Exploratory correlation analyses

The mean score of the LLAS was unrelated to the sum score of the ICAR (*r* = −0.10, *p* = 0.16). Regarding the LLAS dimensions, Seeking Feedback (*r* = 0.02, *p* = 0.75) and Developing Systematically (*r* = −0.04, *p* = 0.58) were unrelated to the sum score of the ICAR. However, similar to our results in Study 2b, Developing Leadership was negatively correlated with the sum score of the ICAR (*r* = −0.18, *p* < 0.01).

### Study 3: Discussion

In Study 3 we further examined the construct-related validity of the LLAS among a field sample of leaders. Again, we found a good model fit for the three-dimension structure of the LLAS. Furthermore, scores on the LLAS were positively related to extraversion and conscientiousness but unrelated to openness to experience. Moreover, scores on the LLAS were unrelated to emotionality, honesty-humility, and agreeableness. Additionally, at an aggregate level, leadership learning agility was unrelated to cognitive ability. Similar to the results in Study 2b, we found a negative relation between the Developing Leadership dimension and cognitive ability.

## Overall discussion

### Main study findings

We developed the Leadership Learning Agility Scale (LLAS), which comprises 18 items and three dimensions. The LLAS showed adequate internal consistency and a consistent factor structure across three independent samples of workers and leaders. Leadership learning agility was positively related to achievement motivation, extraversion, and conscientiousness, but unrelated to openness to experience, emotionality, honesty-humility, and agreeableness. At an aggregate level, leadership learning agility was also unrelated to cognitive ability.

### Theoretical implications

We contribute to the leadership development and talent management literature by responding to calls for further conceptualizing learning agility, and for developing psychometrically sound measures to assess it (e.g., DeRue et al., 2012a; De Meuse, 2017). Although learning agility is seen as a valid predictor construct that relates to growth in leader effectiveness, academic research on this subject is scarce; prior studies included mostly practitioner-oriented articles with questions regarding the conceptualizations and the interpretation of the research data (Bouland-van Dam et al., 2021). Specifically, to stimulate future research we developed a new scale to measure leadership learning agility that is freely accessible and easy to administer. Moreover, we provided initial evidence of the reliability and construct validity of our new scale.

We tried to capture the full leadership learning agility domain with the LLAS. Our results showed that leadership learning agility comprises a Developing Leadership, Seeking Feedback, and Developing Systematically dimension. Hence, the motivational (i.e., and not the aptitude) component in our construct definition was most dominant. In hindsight, if we look at our results, the initially theorized Learning Through Social Interaction dimension relates to, or fits into Developing Leadership; one has to socially interact with others in order to grow in related leader competencies (Larsson et al., 2006). In contrast, the initially theorized Knowing Oneself dimension seems more independent due to the relatively inward and thus less behaviorally active attitude associated with internal reflection processes. Thus, leadership learning agility is associated with “outgoing” instead of internally oriented active learning, which is in line with views in prior research (e.g., De Meuse et al., 2010). Based on these results, leadership learning agility, as measured with the LLAS, refers to the willingness to learn from social experiences, and the drive to apply those lessons in new and challenging leadership roles.

Regarding its nomological net, and from a theoretical standpoint, leadership learning agility most probably relates to, but is distinguishable from constructs such as social skills, proactive behavior at work, and political skills. To illustrate, leadership learning agility relates to social sensitivity (i.e., a social skill, defined as the ability “to read” social rules and norms; Riggio and Reichard, 2008), proactivity (i.e., a trait that underlies active interpersonal behaviors; De Vries et al., 2016), or social astuteness (i.e., a political skill, defined as the ability to adjust to diverse social situations; Ferris et al., 2005). In contrast to these conceptualizations, however, leadership learning agility has a strong motivational component. Moreover, following early learning agility researchers (e.g., Spreitzer et al., 1997; Lombardo and Eichinger, 2000; Eichinger and Lombardo, 2004), and in agreement with for instance the trait approach in emotional intelligence research (e.g., O'Connor et al., 2019), leadership learning agility is a stable trait rather than an ability (Côté, 2014), as it describes typical behavioral and motivational tendencies to learn from social experiences at work and the drive to apply those lessons in leadership roles.

### Unexpected findings

Interestingly, leadership learning agility was unrelated to openness to experience. Although openness to experience, or to engage in idea-related endeavors, might relate to having positive attitudes toward learning experiences in general (Ashton and Lee, 2007), this does not imply that an individual *actively seeks* experiences to learn from. For this active learning attitude, extraversion and conscientiousness might be more important attributes (Ashton and Lee, 2007), which is supported by the strong associations of these specific personality dimensions with the LLAS and its dimensions in our research.

Next, although all LLAS dimensions were positively related to achievement motivation, Developing Leadership and Developing Systematically showed a stronger correlation with achievement *behaviors* than Seeking Feedback. This latter finding surprised us, because seeking feedback is considered a part of achievement behaviors (Smith et al., 2020). When comparing the feedback items of the LLAS and the AMM, we noticed that the AMM feedback items are categorized in achievement *thoughts*, which explains the weaker correlation with Seeking Feedback. Also, the items to measure AMM *behaviors* are more closely related to Developing Leadership and Developing Systematically rather than to Seeking Feedback. In sum, the LLAS dimensions relate to the drive to attain a high standard of performance. Consequently, the LLAS comprises a strong motivational component. This is supported by our factor analytic results: Learning Through Social Interaction and Knowing Oneself failed to load on separate factors.

Finally, in Studies 2 and 3, the Developing Leadership dimension was negatively related to cognitive ability. As explained in the discussion of Study 2b, the negative correlation suggests a compensatory effect of ability and motivation (Van Iddekinge et al., 2018). It seems likely that individuals with lower cognitive ability tend to compensate for their lower cognitive ability by seeking opportunities to engage and put effort in those activities to develop oneself within a social context and toward leadership roles. Although cognitive ability is important for success in leadership roles and specifically for jobs that require the handling of cognitively complex domains (Antonakis et al., 2020), previous studies have shown that motivation-related constructs have stronger validity in predicting educational and work outcomes for individuals with lower cognitive ability compared to individuals with higher cognitive ability (e.g., Fiori, 2015; Light and Nencka, 2019). Moreover, it has been suggested that if the criterion for leaders’ success relates to the domain of interpersonal interaction, leaders must be smart but not too smart (Antonakis et al., 2020).

### Implications for practice

Our study contributes to practice by providing a new scale to measure leadership learning agility. Rather than deriving it from a diverse set of personal attributes such as personality or personal values, which occurs in consulting practice (De Meuse, 2017), we developed a scale to exclusively measure leadership learning agility. We invite practitioners to use the LLAS in their leadership development programs, to learn more about the scale’s practical usage and its criterion-related validity in specific organizational contexts.

The LLAS is suited to administer among workers, to enable focused talent development discussions, and to gain insight in one’s active learning orientation and the drive to develop toward leadership roles. Moreover, as active learning is applied in practice by trying out several (managerial) roles to increase one’s development through diverse experiences (e.g., McCauley et al., 1994), our scale could be used to focus talent management discussions on both an individual and organizational level (Day, 2000). By using the scores on the LLAS, one’s drive and active learning orientation toward leader development are made explicit, and developmental interventions could be focused on individual skills development that ultimately creates organizational value.

### Limitations of this study and future research directions

First and foremost, although we followed established scale development and validation procedures, we did not provide evidence of the criterion-related validity of the LLAS. Therefore, we cannot support prior statements in the literature that refer to learning agility as a valid predictor of leader potential and performance (e.g., De Meuse, 2017). We acknowledge the importance of studying the criterion-related validity of the LLAS, but we believe that the conceptual refinement and subsequent scale development was the first step to advance the field. To provide further evidence of a higher-order model, succeeding criterion-related validity research can examine whether leadership learning agility predicts specific criteria as well as, or better than, its stand-alone dimensions (Johnson et al., 2012). Moreover, based on our conceptualization, leadership learning agility most probably relates to constructs such as social skills, proactivity, and specific leadership styles. For instance, leadership learning agility (i.e., the overall construct) most probably relates to a repertoire of effective leader behaviors such as transformational and contingent reward leadership (Howell and Avolio, 1993; Bass et al., 2003), as seeking feedback, systematic development, and the drive to develop oneself within a leadership context are associated with (developing toward) effective leader behaviors. As studying such relationships is beyond the scope of this manuscript, future research could examine the nomological net in which leadership learning agility is embedded in, in order to support these theoretical standpoints.

Additionally, regarding our conceptualization of leadership learning agility and our subsequent empirical results, we approached it mainly from a motivational perspective. One might consider this a limitation of the current study, as we did not measure individuals’ *ability* to learn from social experiences. However, prior research has shown that compared with motivation, general cognitive ability tends to be a better predictor of maximum (e.g., short-term) performance. In contrast, motivation is relatively more important than ability when predicting typical (e.g., long-term) performance (Van Iddekinge et al., 2018). Hence, leadership learning agility, as measured by the LLAS, most probably is a weaker predictor of short-term performance but a stronger predictor of long-term job performance. Consequently, our approach regarding a motivational perspective toward conceptualizing leadership learning agility seems valid, although future criterion-related research is required to support this view. In addition, when selecting and developing (future) leaders, besides motivational predictors such as leadership learning agility, cognitive ability should be assessed as well, as this is an important yet dissimilar predictor of job performance and leader effectiveness (e.g., Hoffman et al., 2011; Van Iddekinge et al., 2018).

Finally, although we did administer the LLAS among our population of interest, our third sample was range restricted. The high participant dropout rate (i.e., 32%) and the fact that we did not have to exclude any participants based on failed attention checks imply that the participants show a specific personality profile. Indeed, this is supported by the average HEXACO scores: we observed relatively high mean scores on honesty-humility, extraversion, conscientiousness, and openness to experience, compared with values in prior research (Ashton and Lee, 2009). On the other hand, these scores might just reflect a typical leader personality profile (Judge et al., 2002).

### Conclusion

We provided a new scale to measure leadership learning agility that can be applied in both research and practitioner settings. In contemporary organizations, we need to select and develop those leaders that are most effective in adjusting to changing business environments, and for this, learning agility is crucial. We hope that our study stimulates other scholars to advance the learning agility research field.

## Data availability statement

The raw data supporting the conclusions of this article will be made available by the authors, without undue reservation.

## Ethics statement

The studies involving human participants were reviewed and approved by School of Business and Economics Research Ethics Review Board (SBE RERB). The patients/participants provided their informed consent to participate in this study.

## Author contributions

SB-v contributed to conception, design, organizing the database, and writing of all studies within this manuscript, as well as performing all statistical analyses; thus SB-v is considered the first author. JO was the first reviewer of conception, design, and writing of all studies; thus, JO is considered the second author. PJ was the last reviewer of conception, design, and writing of all studies; thus PJ is considered the third author. All authors contributed to manuscript revision and read and approved the submitted version.

## Conflict of interest

The authors declare that the research was conducted in the absence of any commercial or financial relationships that could be construed as a potential conflict of interest.

## Publisher’s note

All claims expressed in this article are solely those of the authors and do not necessarily represent those of their affiliated organizations, or those of the publisher, the editors and the reviewers. Any product that may be evaluated in this article, or claim that may be made by its manufacturer, is not guaranteed or endorsed by the publisher.

## Supplementary material

The Supplementary material for this article can be found online at: https://www.frontiersin.org/articles/10.3389/fpsyg.2022.991299/full#supplementary-material

## Footnotes

^1^Note that we have followed the ethical guidelines of the "SBE Research Ethical Review Board" (i.e., School of Business and Economics), Vrije Universiteit Amsterdam.
