# Supplementary material for: Development and validation of the leadership learning agility scale
Source: Front Psychol. 2022 Dec 23;13:991299. doi: 10.3389/fpsyg.2022.991299 (PMC9817000; doi:10.3389/fpsyg.2022.991299)
Supplement: Supplementary file 1 [file Table_1.DOC]

Table 1 Online Supplemental Material

*The Leadership Learning Agility Scale and Its Scoring Key*

| Item instruction: “Please think about how you behave at work when responding to each statement.” | |
| --- | --- |
|  | At work, I participate in learning activities (e.g., trainings, workshops) to personally develop. |
|  | At work, I carefully evaluate the feedback I receive from others to learn from it. |
|  | At work, I put effort in trying to develop contrasting influential styles (e.g., taking the lead and empowering others). |
|  | I take part in developmental activities to improve my task- and relational skills at work. |
|  | At work, I conceive feedback as a fundamental tool to my performance improvement. |
|  | I put effort in getting better in influencing others to reach our project goals. |
|  | I self-initiate learning activities to improve my job performance. |
|  | I act upon the feedback I receive from peers to improve my job performance. |
|  | I reflect on how to effectively influence my colleagues in our social interactions. |
|  | I participate in trainings because I want to continue developing at work. |
|  | I examine patterns in my own behavior based on the feedback I receive from co-workers. |
|  | I try to influence the development of my co-workers to attain our project goals. |
|  | I take part in educational programs besides my working activities. |
|  | I take action when a colleague gives feedback to improve my performance. |
|  | I focus on how to effectively lead my peers towards our team goals at work. |
|  | At work, I participate in educational opportunities to further develop. |
|  | I adjust my behavior based on the feedback I receive from colleagues. |
|  | I focus on how to become an influencer in my organization to reach our targets. |
| *Note.* Scoring key: Developing Leadership (items 3, 6, 9, 12, 15, 18); Seeking Feedback (items 2, 5, 8, 11, 14, 17); Developing Systematically (items 1, 4, 7, 10, 13, 16). | |
